# Irinotecan, cisplatin and mitomycin in inoperable gastro-oesophageal and pancreatic cancers – a new active regimen

**DOI:** 10.1038/sj.bjc.6600553

**Published:** 2002-10-07

**Authors:** S Slater, J Shamash, P Wilson, C J Gallagher, M L Slevin

**Affiliations:** Department of Medical Oncology, St Bartholomew's Hospital, London EC1A 7BE, UK

**Keywords:** cisplatin, gastro-oesophageal cancer, irinotecan, mitomycin, pancreatic cancer

## Abstract

Irinotecan, mitomycin and cisplatin all demonstrate activity in gastro-oesophageal cancers. This novel combination was administered to outpatients with previously untreated inoperable gastro-oesophageal or pancreatic cancer, in a 28-day cycle. A total of 26 out of 31 patients with gastro-oesophageal cancer and 12 out of 14 patients with pancreatic cancer have been treated with this combination, and were evaluable for response. The overall response rates for patients with gastro-oesophageal cancer was 42%, with a median survival of 9.5 months. In patients with pancreatic cancer, the overall response rate was 42% with a median survival of 8 months. There was a statistically significant increase in survival between those patients who achieved a stable disease response and those who achieved either a partial response or complete response. The toxicity profiles for both cancers were virtually identical. There were five treatment-related deaths, and a high admission rate (42%). Thus irinotecan, mitomycin and cisplatin is a new combination with activity in inoperable upper gastro-oesophageal cancers, but with a high toxicity profile. Future developments include reducing the dose of irinotecan and number of cycles of therapy to four.

*British Journal of Cancer* (2002) **87**, 850–853. doi:10.1038/sj.bjc.6600553 www.bjcancer.com

© 2002 Cancer Research UK

## 

The treatment for inoperable upper gastro-intestinal malignancies remains unsatisfactory. Single agent treatment with 5-fluorouracil (5FU), anthracyclines or cisplatin render response rates as low as 20% (Douglass, 1985). Combination therapies initially experienced greater success: the original response rate for the standard treatment in Europe, FAMTX (5FU, doxorubicin and methotrexate) was 58% (Klein, 1989). Unfortunately a recent EORTC (European Organisation for Research And Treatment of Cancer) study comparing this regimen to ELF (bolus 5FU, etoposide and folinic acid) and FUP (5FU and cisplatin) reported a dismal response rate of 12% for the former, due at least in part to dose reductions and treatment delays (Vanhoefer *et al*, 2000). There were no significant differences in response rates, toxicity profiles or overall survival.

The current standard therapy in the United Kingdom for patients with gastro-oesophageal cancer, adequate renal function and a reasonable performance status is ECF (epirubicin, cisplatin and continuous infusional fluorouracil), the best results being noted in patients with nodal and hepatic metastases (Webb *et al*, 1997). A randomised trial comparing ECF to FAMTX in patients with advanced oesophagogastric cancer, reported response rates of 45% and 21% respectively, with an increase in the median survival duration from 5.7 to 8.9 months. The ECF regimen also demonstrated better quality of life and less haematological toxicity, compared to FAMTX. The regimen has, however, significant inherent problems: it has a moderately emetic potential, most patients require overnight admission for the cisplatin administration, and a tunnelled venous line is required for the delivery of 5FU. Hair loss is common and problems related to the central line are not infrequent. In addition, the infusional fluorouracil requires a pump change weekly either at hospital or at home by a district nurse.

Alternative regimens are thus being explored: the combination of irinotecan with cisplatin has been attempted by several groups in gastro-oesophageal cancer. In the initial study by Shirao *et al* (1997), cisplatin 80 mg m^−2^ was given on day 1 and irinotecan 80 mg m^−2^ on days 1 and 15, the cycle being repeated every 28 days. Mild diarrhoea was seen in 47% of patients (grade 1+2), which resolved with loperamide: only 4% had severe diarrhoea. Nausea and vomiting were easily controlled with 5HT_3_ antagonists. The response rate in this study was 42% with a median time to response of 36 days. Boku *et al* (1999) used a similar 2-weekly combination in patients with advanced gastric cancer. The overall response rate was 48% in the 44 patients treated, with a median survival of 272 days.

The drug mitomycin C has also been extensively investigated in the treatment of upper gastro-intestinal cancers, as a single agent or in combination, and in both adjuvant and metastatic settings. In the 1970s, the FAM regimen comprising 5FU, doxorubicin, mitomycin reported a response rate of 42% in patients with advanced gastric cancer (Macdonald *et al*, 1980). More recently, the results of the study comparing ECF and MCF (mitomycin, cisplatin, 5FU) demonstrated identical response rates, disease-free survival and overall survival (Andersen *et al*, 1999). Mitomycin is a potential candidate for a combination chemotherapy regimen in gastro-oesophageal cancer.

The aim of this study was to develop a regimen of these three active drugs (irinotecan, cisplatin and mitomycin), in gastro- oesophageal and pancreatic cancers, delivering it in a more convenient outpatient setting, whilst achieving an acceptable toxicity profile and response rate.

## MATERIALS AND METHODS

Patients were entered into the study following referral to the Department of Medical Oncology, St. Bartholomew's Hospital, London, and if they satisfied the following entry criteria: histologically proven, inoperable adenocarcinoma or squamous carcinoma of the oesophagus, adenocarcinoma of the stomach or pancreatic carcinoma and had received no prior chemotherapy; performance status 0–3; creatinine clearance or EDTA clearance greater than 50 ml min^−1^; adequate haematological reserve (i.e. platelets >100×10^9^ l^−1^, white cell count >3×10^9^ l^−1^, haemoglobin >100 g l^−1^ (may be transfused). The study had the approval of the local research and ethics committee and patients were required to give written informed consent.

All patients had baseline blood investigations and a staging CT scan of the chest and abdomen, prior to the initiation of therapy.

### Treatment

The combination regimen adopted by Boku *et al* (1999) used a combination of 70 mg m^−2^ of irinotecan on days 1 and 15 with 80 mg m^−2^ of cisplatin on day 1 every 4 weeks, with acceptable toxicity profiles. We proposed to maintain a 2-weekly regimen, and thus 6 mg m^−2^ of mitomycin was administered on Day 1 only, with 100 mg m^−2^ of irinotecan and 40 mg m^−2^ of cisplatin on Days 1 and 15 of the 28 day cycle. A total of 6 cycles of chemotherapy were planned, with omission of mitomycin for the last 2 cycles.

Patients received oral pre-medication with granisetron 1 mg, dexamethasone 16 mg and frusemide 40 mg. This was followed by a 1 l infusion of normal saline with 10 mmol of potassium and 10 mmols of magnesium sulphate over 1 h. Atropine was administered if required. Irinotecan was administered over 30 min, cisplatin in 1 l of normal saline over 1 h.

Patients were reassessed using CT scanning after three cycles, and if progressive disease identified, they were withdrawn from the study. After six cycles, patients were again scanned.

### Dose modifications

Prior to each chemotherapy cycle, the minimum platelet count and neutrophil count permitted to receive treatment were 100×10^9^ l^−1^ and 1.0×10^9^ l^−1^, respectively. If these counts were not achieved, treatment was delayed 1 week. If after 1 week's delay, the platelet count was greater than 75 and rising, and if the neutrophil count was greater than 1×10^9^ l^−1^, the full dose of treatment was administered. If the platelet count was less than 75, mitomycin was prescribed at 50% of the original dose. If the platelet count was between 30–75, a further 1 week's delay was made, followed by a 50% dose reduction in the mitomycin on all following cycles. If the platelet count was less than 30, after the initial 1 week's delay, mitomycin was omitted on all subsequent courses, and all other drugs reduced by 20%. If patients were admitted with Grade 3–4 infections, the doses of all drugs were reduced by 20% for future cycles. If the creatinine increased by more than 15%, a creatinine clearance was calculated. If this proved to be less than 40 ml min^−1^, cisplatin was omitted from subsequent courses.

### Assessment of response

Responses to treatment were defined according to World Health Organisation (WHO) criteria. Patients were then seen regularly in clinic. Survival from the date of treatment was analysed. Performance status (PS) was also recorded, according again to the WHO criteria.

### Patient characteristics

From January, 1999 to March, 2001, 31 patients with previously untreated inoperable gastro-oesphageal cancer and 14 patients with pancreatic cancer were entered into the study. Their characteristics are summarised in Table 1a and b

**Table 1:**
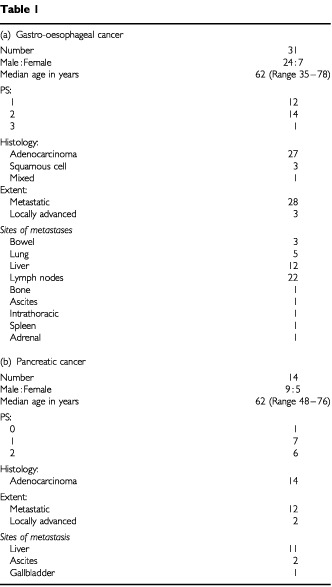

. The PS was not documented in four patients.

## RESULTS

Twenty-six out of 31 patients with gastro-oesophageal cancer and 12 out of 14 patients with pancreatic cancer were evaluable for response. The results are shown in Table 2a and b

**Table 2:**
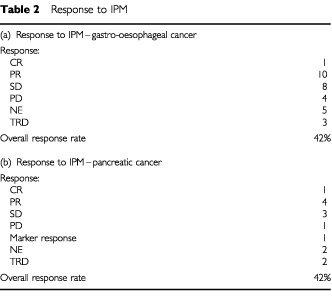
Response to IPM

. The median number of cycles administered in gastro-oesophageal and pancreatic cancer patients was 2.5 and 2.25, respectively (range 0.5–6).

All the data was analysed on an intention to treat basis.

### Gastro-oesophageal cancer

Complete remission was achieved in one patient, who had adenocarcinoma of the oesophagus and metastases to the coeliac lymph nodes and the diaphragmatic crura. Of the 10 partial responses in gastro-oesophageal cancer, nine were in patients with metastatic disease.

Response rates relative to disease site gave the following results: in the two patients with nodal disease, complete response (CR) was achieved in one, stable disease (SD) in the other. In the 10 patients with hepatic disease, partial response (PR) was documented in five, SD in three, and progressive disease (PD) in two.

A PR response was reported in two out of the three patients with peritoneal involvement, with PD being noted in the third.

Four out of the six patients with gastro-oesophageal cancer stable disease after IPM, and seven out of the nine in whom a response to IPM had been achieved, were progression free at 6 months.

Response according to performance status was also analysed. Four out of the twelve patients with PS 1 achieved either a PR or CR response, compared with 6 out of 9 patients with PS of 2–3.

### Pancreatic cancer

CR was achieved in one patient with cancer of the pancreatic head and liver metastases. None of the three patients with pancreatic cancer that was stable after IPM, had progression-free disease by 6 months, compared to four out of the five who responded to IPM.

In pancreatic cancer, responses were noted in four out of the seven patients with PS 0–1, and in one out of the three patients with PS 2–3.

### Non-evaluable patients

In total, the responses to IPM were not evaluable in seven patients – five with gastro-oesophageal and two with pancreatic cancer. All these patients had met the study entry criteria. The median PS of the non-evaluable patients was 2.

Of these seven patients, one completed two cycles of IPM, but did not attend further appointments at this Hospital, and died on day 130 of an unknown cause. Six patients withdrew after receiving day 1 of the first cycle of IPM, four because of toxicity.

### Toxicity data

Data on at least 125 courses of IPM was available for toxicity assessment, and is shown in Table 3a and b

**Table 3:**
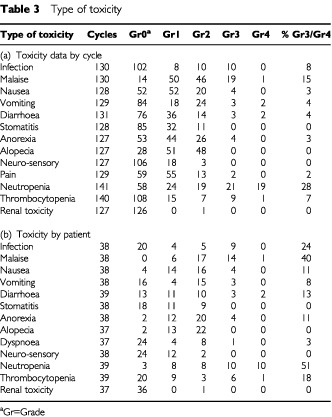
Type of toxicity

. Twenty-six patients had no admissions, 13 patients had one admission, four had two admissions, and two patients had three admissions.

Overall the dose intensity of the IPM regimen was 97.2% (96.9% for irinotecan and cisplatin and 97.7% for mitomycin).

## DISCUSSION

The results of this preliminary study have demonstrated that the combination of irinotecan, mitomycin and cisplatin (IPM) produces an encouraging overall response rate of 42% and a median survival of 9.5 months in patients with inoperable gastro-oesophageal cancer. The response rate compares favourably with the reported response rate of 45% in the multi-centre trial of ECF (Webb *et al*, 1997). In contrast to ECF where a higher response rate was noted in patients with nodal disease, no difference in the response rate to IPM was noted between patients with lymph node, hepatic or peritoneal metastases. Six out of nine patients with PS of 2–3 responded to IPM, leading to the observation that a poor initial performance state should not deter enrolling such patients in studies.

In pancreatic cancer, the National Institute for Clinical Excellence (NICE) has recommended considering single agent gemcitabine as first line therapy with a reported median survival of 5.6 months (Burris *et al*, 1997). In the limited cohort of 14 patients with inoperable pancreatic cancer, the response rate to IPM was 42%, with a median survival of 8 months. IPM has thus shown promise, comparing favourably with the PEF-G regimen (cisplatin, epirubicin, gemcitabine and continuous infusional 5FU), with its objective response rate of 58% in 43 patients (Reni *et al*, 2001). The number of patients treated in this study, however, is low, and would appear to reflect the poor referral rate to medical oncologists and the general pessimism with which chemotherapy is viewed in pancreatic cancer.

The median survival in responders was 10.9 months (95% c.i. 6.7, 15.2: range 5.3–17.6 months) for patients with gastro-oesophageal cancer, and 13.8 months (95% c.i. 2.9, 24.7: range 8–32.6 months) in patients with pancreatic cancer, compared with median survival in the non- responders of 9.5 months (95% c.i. 1.7, 17.2: range 2.2–19.7 months) and 5.4 months (95% c.i. 2.0, 8.8: 1.0–7.3 months) respectively. Thus in patients with pancreatic cancer, those who responded to IPM had a significantly better survival than those who did not (*P*=0.0018).

The median survival in the evaluable (*n*=26) *vs* the non-evaluable (*n*=5) patients with gastro-oesophageal cancer was also found to be statistically different: 10 months *vs* 2.3 months (*P*<0.001).

The results in overall responders versus non-responders and evaluable *vs* non-evaluable patients were, however, hampered by the limited numbers of patients recruited thus far: there were only two non-evaluable patients with pancreatic cancer. The data is worthy of consideration, but must be viewed with caution.

Performance status did not appear to influence outcome to therapy: in patients with both gastro-oesophageal and pancreatic cancers, there was no statistical significant difference in the median survival, relative to the PS.

The IPM regimen had moderate toxicity, with the main side effect being myelosuppression. There were no cases of nephrotoxicity with this regimen, despite concerns over the day case administration of cisplatin with limited intravenous hydration.

However, there were five treatment-related deaths: three from sepsis, one from multi-organ failure, and one from bowel perforation. In total, there were 27 inpatient episodes involving 19 patients: 12 inpatient admissions for infections, seven of these in the presence of neutropenia. There were four admissions for vomiting, three in the same patient. Two admissions were for general malaise, one for a deep vein thrombosis, one for gastro-intestinal bleed in the presence of a normal blood count and clotting, and five admissions in two patients for undocumented reasons. Two patients were admitted in order to administer chemotherapy. Eleven of the original 45 patients failed to complete the first two cycles of IPM, because of toxicity (24%).

There was also a high rate of non-evaluable patients (7 out of 45: 16%). As has been noted the median performance status in this group was 2, and the high drop-off rate may reflect the poor general health in these patients.

Preclinical trials in gastric cancer combining mitomycin and irinotecan suggested synergism. On the basis of this, a trial using 5 mg m^−2^ of mitomycin and 100–150 mg m^−2^ of irinotecan every 2 weeks was initiated, and preliminary results demonstrated a response in 5 out of 10 patients (Handa *et al*, 1999). The current treatment scheduling of IPM has thus been fortuitous: recently presented phase 1 data on irinotecan and mitomycin shows that a modulatory/synergistic relationship may exist between these two drugs, with mitomycin inducing topo-isomerase 1 gene expression (Villalona-Calero *et al*, 2001). A number of trials have confirmed the role of irinotecan and cisplatin in the treatment of previously untreated patients with metastatic adenocarcinoma of the oesophagus and stomach, with reported response rates of 53% and 57%, respectively (Ilson *et al*, 1999). Thus the pharmacological basis for this trial appears sound.

This study has demonstrated the efficacy and potential of IPM, a non-fluorouracil-based treatment, in upper gastro-oesophageal and pancreatic cancers. Patients with pancreas cancer had a significant improvement in the median survival if a response to IPM was achieved. The treatment has, however, resulted in a high toxicity rate: a treatment-related death rate of 11%, and an admission rate of 42%. Future adaptations to this regimen should take into account the difficulties experienced, and reduce the dose of irinotecan to 70 mg m^−2^, and limit the number of cycles to four. Other considerations should also be into exploring the addition of 5FU in this regimen. To maintain the important outpatient element, 5FU could be administered in its oral format, capecitabine.
